# *In vivo* properties of the disaggregase function of J‐proteins and Hsc70 in *Caenorhabditis elegans* stress and aging

**DOI:** 10.1111/acel.12686

**Published:** 2017-10-10

**Authors:** Janine Kirstein, Kristin Arnsburg, Annika Scior, Anna Szlachcic, D. Lys Guilbride, Richard I. Morimoto, Bernd Bukau, Nadinath B. Nillegoda

**Affiliations:** ^1^ Leibniz‐Institute for Molecular Pharmacology (FMP) 13125 Berlin Germany; ^2^ Center for Molecular Biology (ZMBH) Heidelberg University 69120 Heidelberg Germany; ^3^ Department of Molecular Biosciences Rice Institute for Biomedical Research Northwestern University Evanston IL 60208 USA; ^4^ German Cancer Research Center (DKFZ) 69120 Heidelberg Germany

**Keywords:** *C. elegans*, Hsp40, J‐protein, longevity, metazoan, network, protein disaggregation, stress recovery

## Abstract

Protein aggregation is enhanced upon exposure to various stress conditions and aging, which suggests that the quality control machinery regulating protein homeostasis could exhibit varied capacities in different stages of organismal lifespan. Recently, an efficient metazoan disaggregase activity was identified *in vitro,* which requires the Hsp70 chaperone and Hsp110 nucleotide exchange factor, together with single or cooperating J‐protein co‐chaperones of classes A and B. Here, we describe how the orthologous Hsp70s and J‐protein of *Caenorhabditis elegans* work together to resolve protein aggregates both *in vivo* and *in vitro* to benefit organismal health. Using an RNAi knockdown approach, we show that class A and B J‐proteins cooperate to form an interactive flexible network that relocalizes to protein aggregates upon heat shock and preferentially recruits constitutive Hsc70 to disaggregate heat‐induced protein aggregates and polyQ aggregates that form in an age‐dependent manner. Cooperation between class A and B J‐proteins is also required for organismal health and promotes thermotolerance, maintenance of fecundity, and extended viability after heat stress. This disaggregase function of J‐proteins and Hsc70 therefore constitutes a powerful regulatory network that is key to Hsc70‐based protein quality control mechanisms in metazoa with a central role in the clearance of aggregates, stress recovery, and organismal fitness in aging.

## Introduction

Aberrant proteins generated by proteotoxic stresses such as heat shock are actively sequestered into aggregates in cells, which is thought to initially provide a cytoprotective role (Kopito, 2000; Escusa‐Toret *et al*., 2013). Persistence of aggregates, however, acquires toxic properties at the cellular level (Lee *et al*., 2004; Ren *et al*., 2009) and is associated with aging and devastating degenerative diseases in humans (Hipp *et al*., 2014). In metazoa, protein disaggregation is facilitated by a molecular machinery comprising of Hsp70, J‐proteins, and nucleotide exchange factor (NEF) Hsp110 (Nillegoda & Bukau, 2015). Substrate targeting of the metazoan Hsp70‐based disaggregase is driven by J‐proteins that concomitantly interact with both substrates and Hsp70 partner proteins. Individual and mixed‐class (A+B) canonical J‐protein complexes promote specific or broad‐range aggregate targeting, respectively, *in vitro* (Gao *et al*., 2015; Nillegoda *et al*., 2015, 2017). Further, the binding of the J‐domain of J‐proteins to Hsp70 stimulates ATP hydrolysis, allowing substrate capture by Hsp70. ADP release from Hsp70 facilitates ATP rebinding and triggers substrate dissociation (Mayer & Bukau, 2005), promoted by Hsp110, which further boosts the activity of the disaggregase (Shorter, 2011; Rampelt *et al*., 2012).

**Figure 1:**
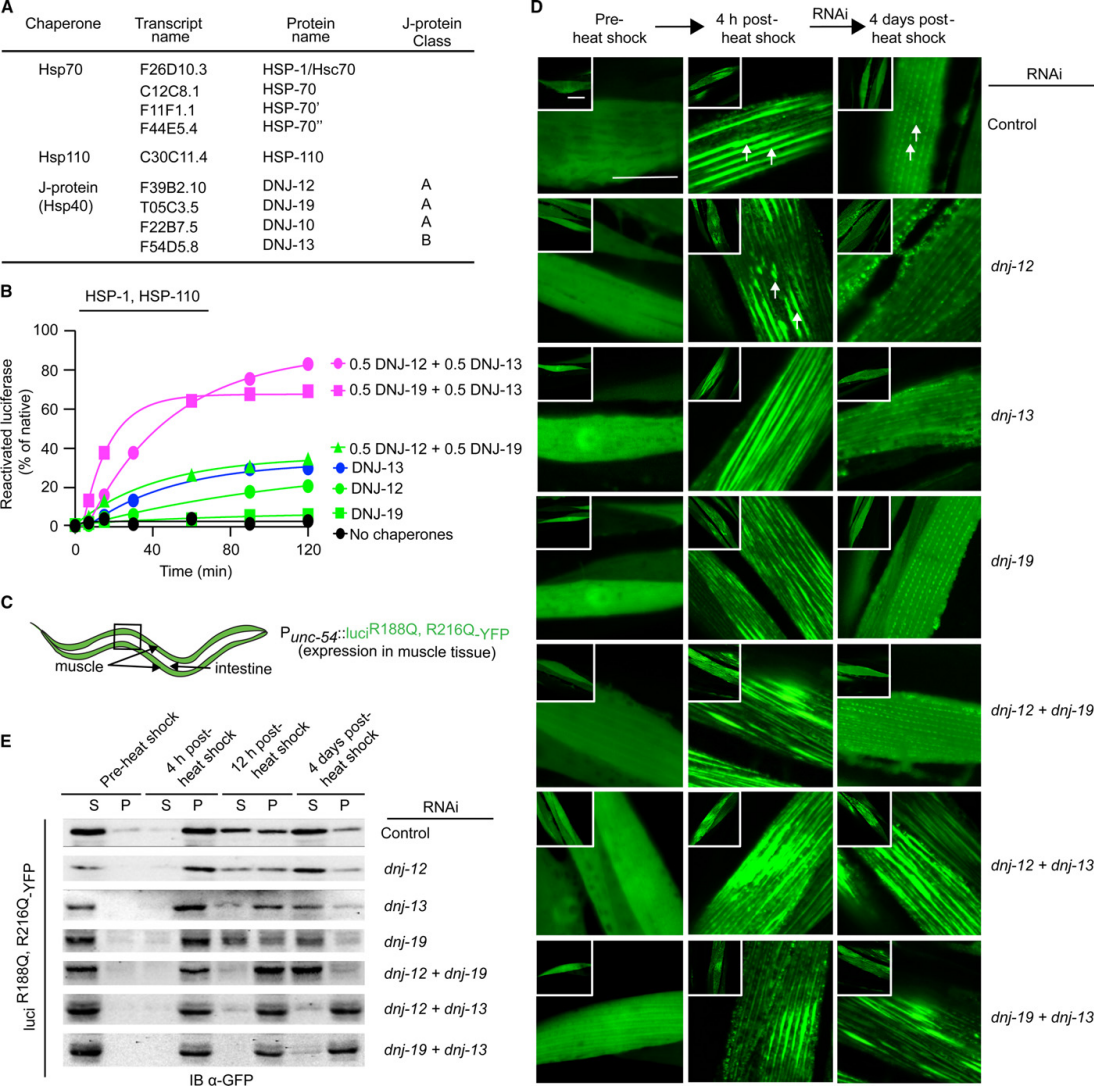
*In vitro* and *in vivo* J‐protein class cooperation. (A) Table summarizing cytosolic and nuclear molecular chaperones from *Caenorhabditis elegans* used in the study. (B) *In vitro* reactivation of preformed luciferase aggregates by HSP‐1, HSP‐110, and the indicated J‐protein(s). Control (absence of chaperones) is depicted in black. Single class A and class B J‐protein containing reactions are depicted in green and blue, respectively. Representative experiment shown; *N* = 3. (C) Cartoon depicting the targeted expression of aggregation‐sensor luci^R188Q,R216Q^‐YFP in *C. elegans*. (D) Fluorescent images of *C. elegans* muscle cells expressing luci^R188Q,R216Q^‐YFP. Single and double RNAi knockdowns (KD) of cytosolic canonical class A and class B J‐proteins indicated in text on right. Left, middle, and right image columns show solubility of luciferase prior to heat shock, 4 h post‐heat shock, and 4 days post‐heat shock. White arrows show luciferase aggregates. Control animals fed with empty RNAi vector L4440 (left column, top row). Inset, one entire muscle cell depicted. Scale bar = 10 μm. *N* = 50 nematodes per condition. (E) Western blots of the supernatant and pellet profiles of luci^R188Q,R216Q^‐YFP in lysates obtained from single and double RNAi knockdown (KD) of cytosolic and nuclear class A and class B J‐proteins in muscle tissue of *C. elegans*. The YFP moiety fused to luci^R188Q,R216Q^ immunoblotted with a cross‐reacting antibody raised against GFP.

The Hsp70 archetype, Hsc70 (nematode HSP‐1 (F26D10.3)), is constitutively expressed. In response to adverse stress conditions, nematode cells induce several other cytosolic homologs of Hsc70, termed HSP‐70 (C12C8.1), HSP‐70’ (F11F1.1), and HSP‐70” (F44E5.4) (Nikolaidis & Nei, 2004; Brehme *et al*., 2014) (Fig. 1A). Constitutive Hsc70 and stress‐induced Hsp70s show relatively similar protein refolding activity and holdase function *in vitro* (Freeman & Morimoto, 1996; Dragovic *et al*., 2006; Tutar *et al*., 2006). It is considered that Hsc70 serves as a multitasking housekeeping chaperone *in vivo* (Bukau *et al*., 2006) whereas inducible Hsp70s have been implicated in stress‐related activities such as promoting cell survival (Kobayashi *et al*., 2000; Xanthoudakis & Nicholson, 2000; Rohde *et al*., 2005). The contribution of constitutive vs. inducible Hsp70 homologs in protein disaggregation/clearance after stress in an *in vivo* setting remains experimentally unexamined.

While *in vitro* evidence has established a role for canonical J‐protein networks in protein disaggregation, whether this occurs at an organismal level has not been determined. Metazoa, such as *Caenorhabditis elegans,* express multiple cytosolic and nuclear J‐protein members of all three classes (A, B, and C) (Kampinga & Craig, 2010; Yook *et al*., 2012; Brehme *et al*., 2014). To date, only canonical J‐protein members of classes A and B are implicated in metazoan protein disaggregation and refolding (Gao *et al*., 2015; Nillegoda & Bukau, 2015; Nillegoda *et al*., 2015, 2017). Canonical J‐proteins belonging to class A contain an N‐terminal J‐domain, a glycine/phenylalanine‐rich flexible linker region, a C‐terminal domain containing a zinc‐finger‐like region (ZFLR), and a dimerization domain. Class B J‐proteins lack the ZFLR, but retain a structurally related C‐terminal domain (Kampinga & Craig, 2010). The N‐terminal J‐domain regulates the Hsp70 ATPase cycle, which facilitates high‐affinity substrate binding (Mayer & Bukau, 2005). The varying amino acid sequence composition at the C‐terminal domains of class A and B J‐proteins dictates substrate specificity (Kampinga & Craig, 2010; Borges *et al*., 2012; Nillegoda *et al*., 2015). *Caenorhabditis elegans* has two canonical class A J‐proteins and a single canonical class B J‐protein in the cytosol (Yook *et al*., 2012). How these different J‐proteins of classes A and B relate and operate with different Hsp70 isoforms *in vivo,* especially in protein quality control processes such as aggregate resolution in response to stress, is poorly understood.

Here, we show that cooperation among canonical class A and B J‐proteins in *C. elegans* forms an *in vivo* network, which is stress responsive and essential for stress survival. Contrary to accepted tenets, this network co‐opts and converts primarily the constitutive Hsc70 chaperone, rather than the stress‐induced Hsp70 variants into a highly regulated disaggregase essential for the clearance of protein aggregates and stress recovery *in vivo*, which is also experimentally supported by *in vitro* data. This defines a fundamental diversification of physiological function among Hsp70 homologs and identifies a key regulatory J‐protein interclass network in metazoan organisms that enhances the protein quality machineries in stress, and during aggregate persistence relevant to degenerative diseases.

## Results

### Cooperation among *C. elegans* class A and class B J‐proteins stimulates *in vitro* protein disaggregation

We first established with purified *C. elegans* chaperones (Fig. S1A, Supporting information) whether the protein disaggregation activities described for the human Hsp70 system with different configurations of canonical class A and B J‐proteins are conserved (Nillegoda *et al*., 2015). Amorphous protein aggregates were preformed by thermal denaturation of firefly luciferase at 45 °C, and mixed with the indicated configurations of the Hsp70 chaperone systems to initiate disaggregation (see Methods). Disaggregation reactions containing the *C. elegans* HSP‐1–HSP‐110 (Hsc70‐Hsp110) system with the respective single J‐proteins (class A, DNJ‐12 or DNJ‐19, green; class B, DNJ‐13, blue; Fig. 1B) resulted in ~5–30% reactivation of heat‐denatured firefly luciferase normalized to the activity of native luciferase (set as 100%), whereas reactions containing both class A and B members simultaneously resulted in the synergistic reactivation of luciferase to a level of ~70–80% (magenta; Fig. 1B). Importantly, the same level of disaggregation activity was not observed using two J‐proteins of the same class (DNJ‐12 + DNJ‐19). Therefore, the *C. elegans* Hsc70‐based disaggregation system containing both class A and B J‐proteins exhibits efficient *in vitro* luciferase aggregate solubilization and enzymatic reactivation. This represents only the second metazoan system that extends our previous report of synergy induced by human mixed‐class J‐protein complexes (Nillegoda *et al*., 2015), and provides the basis for the subsequent *in vivo C. elegans* experiments.

### Analysis of *in vivo* cooperation between class A and B J‐proteins

To establish an *in vivo* role for class A and B J‐protein cooperation in protein disaggregation, we expressed a thermolabile luciferase protein aggregation reporter fused to YFP (luci^R188Q,R216Q^‐YFP) (Gupta *et al*., 2011; Rampelt *et al*., 2012) in the body wall muscle cells of *C. elegans* (Fig. 1C,D) similar to our previous work to identify and characterize HSP‐110, the specialized metazoan NEF that also functions in *C. elegans* protein disaggregation (Rampelt *et al*., 2012). The aggregation propensity of the thermolabile luciferase is indirectly monitored by the fluorescent signal pattern generated by the YFP fluorescent tag located at the C‐terminus of the sensor. A diffuse fluorescent signal corresponds to soluble luciferase while a punctated fluorescent pattern indicates the presence of aggregated luciferase in cells. As expected, the fluorescent images of muscle cells in animals living under unstressed growth conditions (20 °C) showed an even distribution of the YFP signal indicative of soluble, natively folded luciferase (pre‐heat shock, Fig. 1D, top row, left column). However, upon exposure to heat shock at 35 °C for 1 h, followed by a short recovery period of 4 h at 20 °C, previously soluble luciferase distributed into punctated aggregate structures that formed along the myofilaments (Fig. 1D, top row, middle column). These luciferase aggregates dissipated with time and after 4 days of recovery from heat shock, only a relatively small number of puncta with reduced size were visible (Fig. 1D, top row, right column).

In order to study the effects of interclass J‐protein cooperation in resolving protein aggregates during stress recovery, we employed an experimental approach using RNAi to knockdown specific cytosolic class A and B J‐protein members in post‐heat shock animals. Our rationale for this experimental strategy to perform RNAi post‐heat shock treatment ensures that the luciferase aggregates are present uniformly in body wall muscle cells and excludes potential confounding effects on aggregate formation by additional J‐protein activities such as prevention of aggregation (holdase function). Moreover, the recovery period of 4 days ensured that RNAi treatment was sufficient to reduce the mRNA levels of the respective J‐proteins (Fig. S1B, Supporting information). The RNAi knockdown panel consisted of single and double knockdown combinations of DNJ‐12 (A), DNJ‐19 (A), and DNJ‐13 (B) J‐proteins that were confirmed to be localized to the cytosol and nucleus of muscle cells using immunofluorescence (Fig. S1C, Supporting information).

### Class A and B J‐protein networking is essential for aggregate clearance *in vivo*

Animals fed and maintained on *E. coli* carrying empty RNAi vector (control) during the recovery period rapidly resolved the luciferase aggregate load during recovery after heat shock treatment (Fig. 1D, top row, right column). Further, we observed reappearance of the diffused fluorescence signal in the cytosol of muscle cells indicative of the presence of solubilized luci^R188Q,R216Q^‐YFP molecules that are most likely refolded as observed in human cells recovering from heat stress (Gupta *et al*., 2011).

To test the role of class A and B J‐proteins for efficient aggregate resolution *in vivo,* we simultaneously knocked down a single J‐protein from each of class A and class B (Fig. 1D, rows 2‐6, right column). Animals exposed to double knockdowns of either *dnj‐12 *+ *dnj‐13* or *dnj‐19 *+ *dnj‐13* showed persistence of the severe aggregate phenotype. This result corroborates our *in vitro* findings (Fig. 1B) and suggests that mixed A and B class J‐protein cooperation is essential for efficient protein disaggregation in animal tissues. In contrast, animals exposed to either single (*dnj‐12*,* dnj‐13,* or *dnj‐19*) or double of the same‐class (*dnj‐12* + *dnj‐19*) J‐protein knockdown efficiently resolved luciferase aggregates to similar levels observed for wild‐type (control) animals (Fig. 1D, rows 1–5, right column).

The double knockdown of both cytosolic/nuclear class A J‐proteins (*dnj‐12* + *dnj‐19*) should inhibit aggregate resolution as a result of considerable disruption of the interclass J‐protein network with DNJ‐13, the only cytosolic/nuclear *C. elegans* class B J‐protein. We reasoned that the absence of a severe aggregate persistence phenotype during recovery after heat shock in the knockdown of *dnj‐12* + *dnj‐19* is due to the presence of another cytosolic class A J‐protein member with a compensatory activity. We considered a role for *C. elegans* mitochondrial class A J‐protein DNJ‐10, which recent work on protein sorting into cellular organelles showed to be partially retained in the cytosol (Gamerdinger *et al*., 2015). Further, DNJ‐10 is homologous to the mitochondrial DNAJA3 in human cells, which has also been shown to function in the cytosol (Lu *et al*., 2006). Based on these observations, we rationalized that in the absence of DNJ‐12 and DNAJ‐19 the cytosolic fraction of DNJ‐10 compensates for and cooperates with DNJ‐13 to support efficient protein disaggregation. To test this, we perform a triple knockdown of all three class A J‐proteins (*dnj‐12* + *dnj‐19 + dnj‐10*), which resulted in a severe aggregate persistence phenotype in post‐heat‐stressed animals (Fig. S1D, Supporting information). Furthermore, double depletion of *dnj‐10* with *dnj‐13* resulted in drastic aggregation persistent phenotype similar to the rest of the class A+B knockdowns consistent with our speculation that DNJ‐10 integrates into the cytosolic J‐protein network during aggregate clearance (Fig. S1D, Supporting information). Additionally, the same‐class double knockdowns involving *dnj‐10* (*dnj‐12* + *dnj‐10* and *dnj‐19* + *dnj‐10*) resolved luciferase aggregates efficiently comparable to wild‐type animals (Fig. S1D, Supporting information). Thus, the depletion of individual J‐proteins or same‐class J‐protein double depletion does not impair the cellular disaggregation capacity and these treated animals show a recovery from heat shock comparable to the control animals. Unstressed animals (without heat shock) subjected to triple knockdown of all class A J‐proteins (data not shown) or class A+B J‐protein depletions (Fig. S1E, Supporting information) showed no aggregation of luciferase. These results indicate that co‐depletion of mixed‐class J‐proteins are tolerated in the absence of a proteostasis perturbation and do not appear to compromise the housekeeping protein quality control functions. Taken together, our results indicate that interclass J‐protein cooperation is necessary *in vivo* for efficient protein quality control and aggregate clearance after systemic stress in *C. elegans* and reveal an additional level of physiological networking for J‐protein function.

To further validate our fluorescence‐based microscopic observations of luciferase aggregation and resolubilization (Fig. 1D), we analyzed the solubility of the thermolabile luciferase upon heat shock and recovery using a protein sedimentation assay, which separates aggregated from soluble proteins in lysates (Fig. 1E). As expected, prior to heat shock (20 °C), luciferase in the animal lysates appears almost exclusively in the soluble (S) fraction (Fig. 1E) consistent with fluorescence data (Fig. 1D). However, upon heat shock (1 h at 35 °C followed by incubation on different *dnj* gene RNAi plates at 20 °C for 4 h), the soluble fraction of luciferase shifted almost entirely to the insoluble pellet (P) fraction in all RNAi treatments indicative of heat‐induced unfolding and aggregation of luciferase. This observation is consistent with the severe aggregation phenotype observed by fluorescence microscopy 4 h post‐heat shock (Fig. 1D). During the subsequent recovery periods (12 h and 4 days), we observed the reappearance of soluble luciferase in the control and all RNAi‐treated strains except in the class A+B J‐protein double knockdowns (*dnj‐12 + dnj‐13* and *dnj‐19 *+ *dnj‐13*) (Fig. 1E). Overall, the data from our supernatant and pellet assays are consistent with the findings from live imaging analysis (Fig 1D) and support our conclusion that cooperation among mixed‐class J‐proteins is essential for the recovery of stress‐induced protein aggregates.

### J‐protein class collaboration affects polyglutamine containing protein aggregate formation *in vivo*

**Figure 2:**
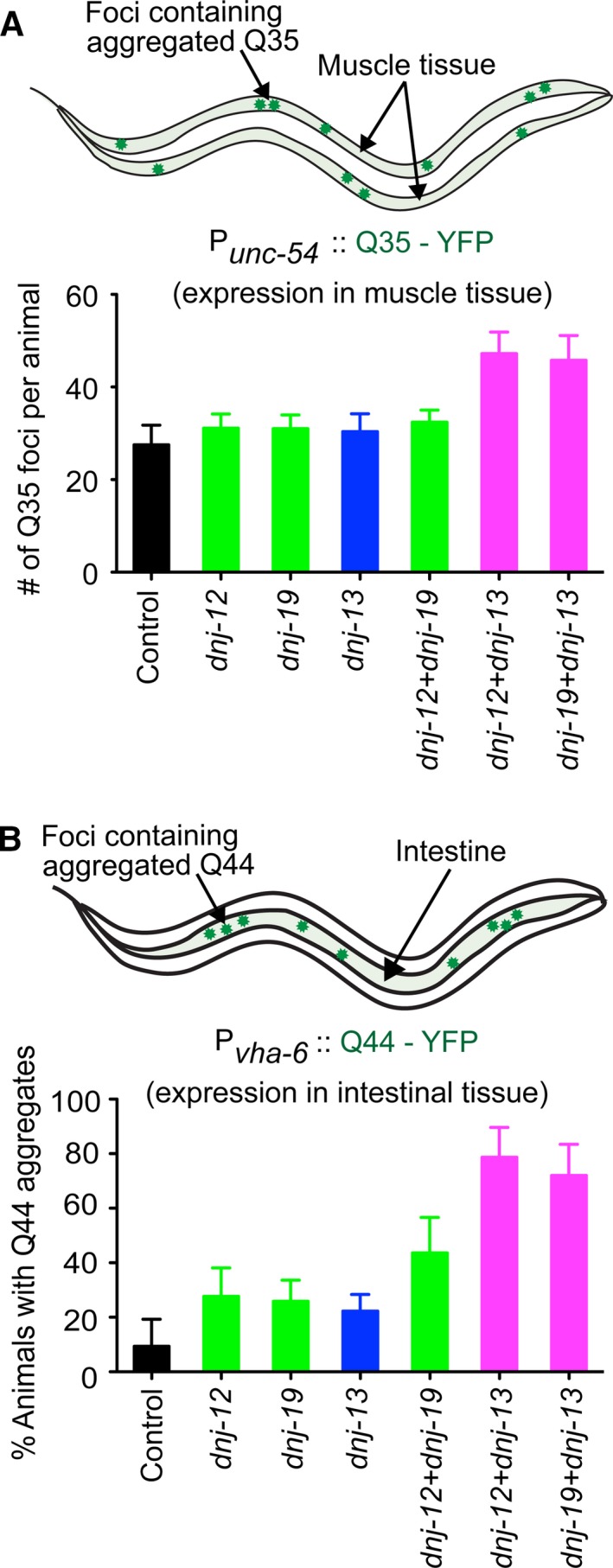
Mixed‐class J‐protein cooperation affects polyQ‐containing protein aggregation in *Caenorhabditis elegans* in a tissue‐independent manner. (A) Polyglutamine Q35‐YFP aggregate quantification at day 5 in muscle tissue, with single and double knockdown of different J‐proteins (see Fig. S2C (Supporting information) for representative fluorescent images). Single and double knockdowns of J‐proteins are depicted in green (class A), blue (class B), and magenta (class A+B). *N* = 30 animals. *P *< 0.0001 one‐tailed ANOVA. (B) Quantification of Q44‐YFP aggregates in 6‐day‐old nematode intestinal tissue with single or double knockdown of *dnj* genes (see Fig. S2D (Supporting information) for representative fluorescent images). *N* = 20 animals; *P *< 0.0001 one‐tailed ANOVA. Error bars reflect mean ± SD.

To investigate whether mixed‐class J‐protein collaboration is required for the clearance of other types of protein aggregates, we employed a C‐terminally YFP‐tagged polyQ protein containing 35 glutamines (Q35) that generates insoluble aggregates in muscle tissue in an age‐dependent manner without a heat shock trigger (Fig. S2A,B, Supporting information) (Morley *et al*., 2002). Animals subjected to mixed‐class (A+B) J‐protein double knockdowns showed increased numbers of Q35 aggregates per animal compared to single or double same‐class knockdowns (Fig. 2A). The representative fluorescent images used in aggregate quantification are shown in Fig. S2C (Supporting information). Similar results were also observed in animals expressing polyQ protein, Q44, in the intestine (Fig. 2B, see Fig. S2D (Supporting information) for representative fluorescent images used in aggregate quantification). These observations for polyQ protein aggregation are consistent with the results obtained with heat‐aggregated luciferase (Fig. 1D). Taken together, this suggests that interclass J‐protein networking is important for the efficient resolution of diverse types of protein aggregates in a tissue‐independent manner. The essentiality of the J‐protein collaboration for efficient targeting of Hsp70‐based disaggregases to different aggregate types underscores the *in vivo* relevance of this co‐chaperone network broadly to diverse protein conformational diseases.

### Stress‐responsive regulation of class A and class B J‐proteins

**Figure 3:**
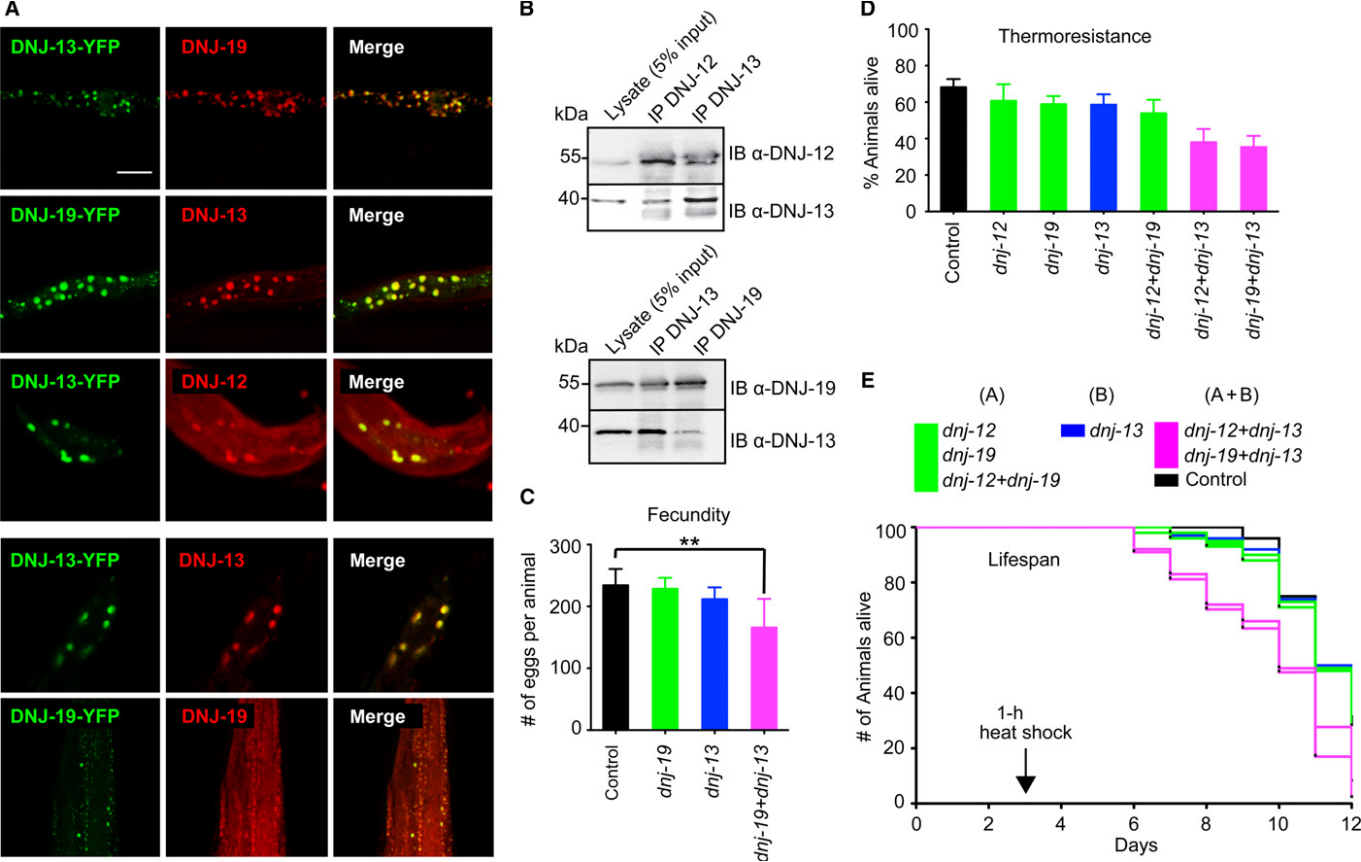
Mixed‐class J‐protein cooperation is stress responsive and vital for organismal health and viability. (A) Co‐localization of immunofluorescence (yellow) of DNJ‐12 (A), DNJ‐13 (B), or DNJ‐19 (A) using Alexa Fluor 594‐labeled antibodies (red), and stably expressed C‐terminal YFP‐tagged (green) DNJ‐13 (B) or DNJ‐19 (A) in *Caenorhabditis elegans* after 4 h recovery at 20 °C post‐heat shock. Fluorescent images depict the head region of nematodes. Scale bar = 20 μm. Bottom two panels show the specificity of antibody recognition for DNJ‐13 and DNJ‐19. (B) DNJ‐12, DNJ‐13, DNJ‐19 co‐immunoprecipitate from non‐heat shocked lysate. IP, immunoprecipitation; IB, immunoblot (see Fig. S2G (Supporting information) for the controls for co‐IPs). (C) Fecundity upon single or double knockdown of *dnj* genes. Class A+B double knockdown, magenta; same‐class single or double knockdowns, class A, green; class B, blue. Heat shock 35 °C, for 1 h at L4 stage on day 3. Knockdown of *dnj‐12* shows embryonic lethality. Eggs laid per animal (*N* = 10 animals). Unpaired Student's t‐test with Welch's correction **P = 0.0012. (D) Thermoresistance, % survival on day 6 after prolonged 35 °C heat shock (6 h). *N* = 100 animals. One‐tailed ANOVA *P* < 0.0001. (E) Percent viability of nematodes with single and same‐class double knockdown (green (A), blue (B)) or mixed‐class double KD (magenta) of *dnj* genes. Heat shock 35 °C, 1 h at L4 stage on day 3. *N* = 100 animals. Error bars reflect mean ± SD.

We next explored whether proteotoxic stress affects the class A and B J‐protein members at the mRNA and protein levels in *C. elegans*. In animals exposed to heat shock, the levels of *dnj‐12*,* dnj‐19,* and *dnj‐13* mRNA were induced by ~2‐fold in 4‐day‐old young adults (Fig. S2E, Supporting information). In contrast, older animals (10 days old) exhibited a diminished induction of these cytosolic J‐protein mRNA levels upon heat shock. This is in agreement with previous reports showing a similar mRNA induction pattern for *C. elegans* Hsp70s and a collapse of heat shock response and proteostasis during nematode aging (Ben‐Zvi *et al*., 2009; Labbadia & Morimoto, 2015). To assess J‐protein dynamics at the protein level, we employed *C. elegans* strains expressing C‐terminally YFP‐tagged DNJ‐13 and DNJ‐19 and generated antibodies to immunostain endogenous DNJ‐12, DNJ‐13, and DNJ‐19. Each of the J‐protein antibodies was shown to exhibit specificity and did not cross‐react with other cytosolic members of the J‐protein family (Fig. S2F, Supporting information). Using these tools, we observed that all three J‐proteins are diffused in the cytosol and nuclei of muscle cells of control (unstressed) animals (white arrows, Fig. S1C, Supporting information). However, upon heat shock, these class A and B J‐proteins become co‐localized (yellow) into predominantly cytosolic foci possibly comprised of aggregates with misfolded endogenous proteins suggesting an altered functional interaction in response to proteotoxic stress (Fig. 3A, merged). To determine whether the co‐localization of class A or B J‐proteins upon heat shock corresponds to prior interactions in the pre‐heat shock state, we performed immunoprecipitation assays from lysates of unstressed animals (Figs 3B and S1G, Supporting information) and observed the association of the canonical class A or B J‐proteins, consistent with our previous biochemical observations with purified human J‐proteins (Nillegoda *et al*., 2015). Direct evidence showing the existence of mixed‐class J‐protein complexes in unstressed human cells was recently reported (Nillegoda *et al*., 2017) further consolidating our co‐immunoprecipitation‐based findings. This suggests that J‐protein complexes may also function in different housekeeping activities that may not be essential or linked to proteostasis, but increase with stress, implying interclass J‐protein interactions, per se, are responsive to proteotoxic stress.

### A and B class J‐protein network is fundamental to physiology and lifespan

We next addressed whether cooperation between class A and B J‐proteins has general physiological roles by examining the effect of RNAi depletions on fecundity, thermoresistance, and lifespan. Animals exposed to heat shock and subjected to class A+B co‐depletion exhibited a ~30% reduction in fecundity relative to animals depleted for either single A or B J‐protein (Fig. 3C). However, unstressed animals subjected to J‐protein depletions including the *dnj‐13* + *dnj‐19* (class A+B) co‐depletion exhibited no effect on fecundity, which is consistent with our previous findings and further supports the notion that interclass J‐protein cooperation is dispensable in the absence of a proteostasis perturbation (data not shown). Likewise, the heat shocked animals co‐depleted for class A+B J‐proteins showed, thermoresistance declined by 50% (Fig. 3D) and lifespan reduced by ~10% (Fig. 3E). Importantly, without the heat shock challenge, lifespan was unaffected in the class A+B co‐depleted animals (Fig. S3A, Supporting information). The RNAi knockdown efficiencies of J‐proteins (whole animals) for these experimental conditions are indicated in Fig. S1B (Supporting information). Notably, the knockdown efficiencies of double RNAi experiments do not significantly differ from the single knockdown efficiencies and reduced the expression of the respective genes between 72 and 92% relative to vector alone controls (Fig. S1B, Supporting information). Overall, these effects underscored the fundamental physiological relevance of the communication among canonical class A and B J‐proteins during recovery from stress.

### Hsc70 is preferentially utilized for post‐stress aggregate clearance *in vivo*

**Figure 4:**
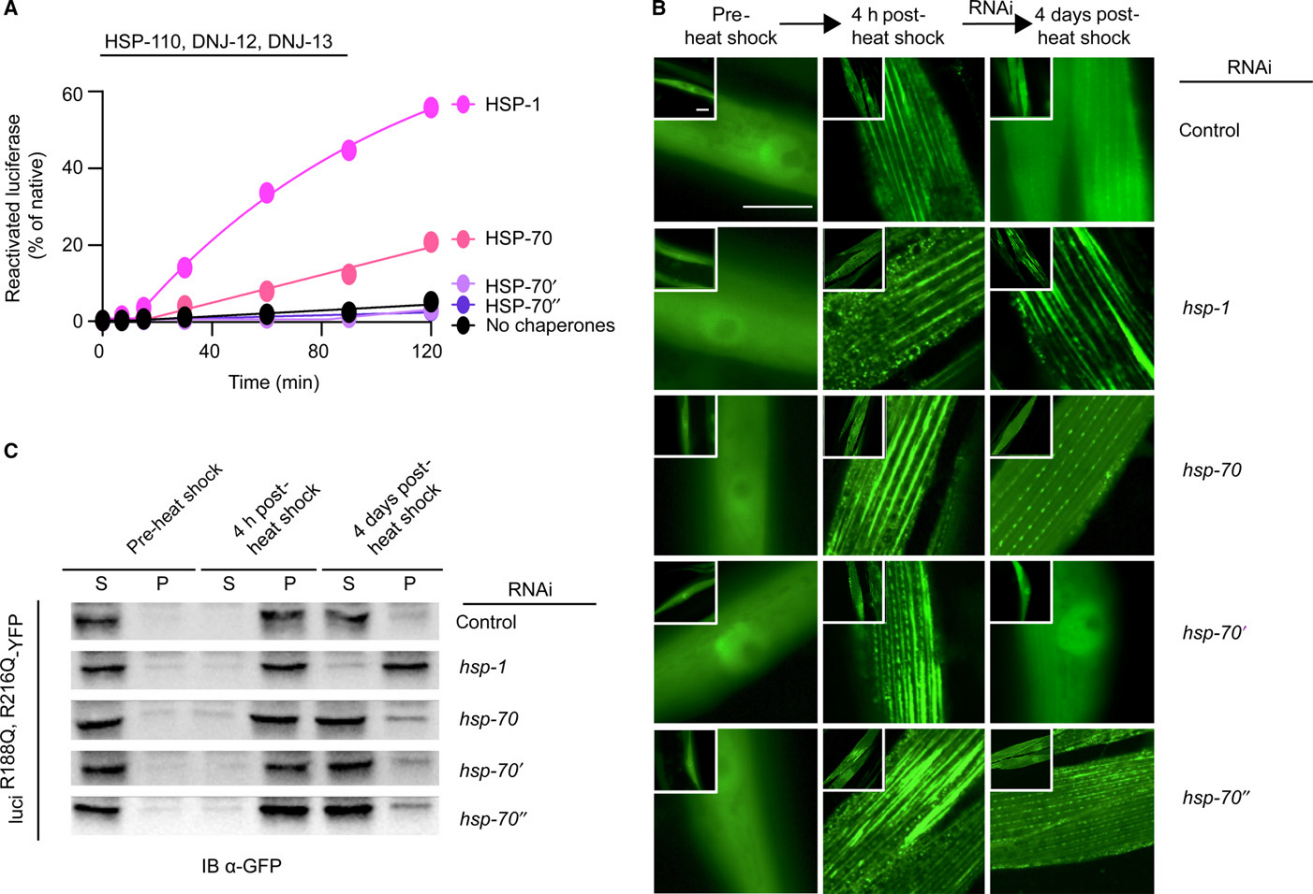
Constitutive HSP‐1 forms the most efficient protein disaggregase *in vitro* and *in vivo*. (A) *In vitro* reactivation of preformed luciferase aggregates (heat‐denatured) with either constitutive HSP‐1 or inducible worm Hsp70 homologs (HSP‐70, HSP‐70’, and HSP‐70”). All disaggregation/refolding reactions contain HSP‐110, DNJ‐12, and DNJ‐13. Control (no chaperones) in black. Representative experiment shown; *N* = 3. (B) Fluorescent images of luci^R188Q,R216Q^‐YFP aggregation in *Caenorhabditis elegans* muscle tissue. Effects of different Hsp70 knockdowns shown prior to heat shock (left column), 4 h post‐heat shock (middle column), and 4 days post‐heat shock (right column). The respective Hsp70 RNAi depletions indicated in text on right. Control, nematodes fed empty RNAi vector L4440 (Top row, left column). Scale bar = 10 μm. *N* = 30 nematodes per condition. (C) Western blots of the supernatant and pellet profiles of luci^R188Q,R216Q^‐YFP in lysates obtained from RNAi knockdown (KD) of cytosolic and nuclear Hsp70 chaperones in muscle tissue of *C. elegans*. The YFP moiety fused to luci^R188Q,R216Q^ immunoblotted with a cross‐reacting antibody raised against GFP.

We compared chaperone systems comprised of the constitutive Hsc70 (HSP‐1) or heat shock‐inducible forms of Hsp70 (HSP‐70, HSP‐70’, or HSP‐70”; Fig. 1A) supplemented with mixed‐class J‐protein complexes and HSP‐110 to determine which member of the Hsp70 family was critical for protein disaggregation (Fig. 4A). Using the *in vitro* protein disaggregation/refolding assay, we find the solubilization and reactivation of heat‐aggregated luciferase are markedly more efficient with HSP‐1 (Fig. 4A). This was demonstrated by comparison with the three stress‐induced homologs, of which only HSP‐70 solubilized aggregated luciferase, at approximately one‐third of the efficiency of HSP‐1 (Fig. 4A). A similar observation was made when we analyzed the contribution of different members of the Hsp70 family in luciferase aggregate solubilization *in vivo*. RNAi knockdown of *hsp‐1* completely abrogated luciferase aggregate solubilization (Fig. 4B), whereas depletion of HSP‐70 and HSP‐70” by RNAi resulted in only moderate aggregate solubilization defects, and HSP‐70’‐depleted animals exhibited no detectable defect. Further, complementary findings from the supernatant‐pellet protein sedimentation assays also show that depletion of *hsp‐1* inhibited the disaggregation of luciferase from aggregates even at day 4 of stress recovery (Fig. 4C). In contrast, the lysates from control animals or those treated with RNAi for the stress‐inducible HSP‐70s (HSP‐70, HSP‐70’, and HSP‐70’’) showed considerable solubilization of stress‐aggregated luciferase at day 4 (Fig. 4C). Consistent with these observations, the thermoresistance assay revealed that RNAi treatment of the constitutive HSP‐1 drastically decreased survival after heat stress whereas depletion of the inducible homologs of Hsp70 had no effect (Fig. S3B, Supporting information). Under these conditions, *hsp‐1* depletion in the absence of heat shock also decreases lifespan, but to a lesser extent (Rampelt *et al*., 2012). Overall, these results establish the functional specificity of the constitutively expressed HSP‐1 chaperone in resolving heat stress‐induced aggregates and reducing the associated cytotoxicity over the stress‐induced HSP‐70 variants, pointing toward a marked functional and physiological specialization for cellular Hsp70 variants.

## Discussion

We recently reported *in vitro* findings that show cooperation between human class A and B J‐proteins via complex formation is essential for efficient Hsp70‐mediated disaggregation of amorphous protein aggregates (Nillegoda *et al*., 2015, 2017). In this study, we show that J‐protein interclass collaboration constitutes the key molecular regulatory network which functions *in vivo* to modulate disaggregation activity in different tissues of the metazoan *C. elegans*. Disruption of the J‐protein class interactions interferes with these fundamental disaggregase activities, leading to decreased thermotolerance, reduced fecundity, shortened lifespan, and persistence of aggregates characteristic of age‐associated degenerative diseases.

The high sensitivity of the J‐protein network to perturbation during stress may be explained by the effects of protein misfolding on cell physiology. For example, heat shock critically depletes ‘free’ chaperone levels in general, even with the compensatory benefits of the heat shock response (Nollen *et al*., 2000; Yu *et al*., 2014). This mimics the J‐protein limiting conditions generated *in vitro* where mixed‐class J‐protein cooperation becomes vital for efficient protein disaggregation (Nillegoda *et al*., 2015). Our data imply that a threshold concentration of each J‐protein class is necessary *in vivo* to provide sufficient mixed‐class molecular interactions to cope with increased aggregation driven by systemic proteotoxic stress. Precisely how this delicate network is able to balance and tolerate double depletion of both cytosolic class A J‐proteins is now explained, and points toward compensatory mechanisms that are either abolished or subverted when mixed‐class J‐proteins are simultaneously depleted. It also suggests that intracellular ratios of individual J‐proteins are themselves major determinants of metazoan protein quality control.

We further show that preventing mixed‐class J‐protein cooperation in *C. elega*ns significantly exacerbates the age‐dependent aggregation of polyQ proteins. In humans, amyloidogenic proteins including polyQ‐containing proteins are directly linked to development of neurodegenerative diseases such as Huntington's, Parkinson's, and spinocerebellar ataxias (MacDonald *et al*., 1993; Padiath *et al*., 2005). Parkinson's‐linked α‐synuclein amyloid fibers are efficiently reversed *in vitro* by specific configurations of the human disaggregation system containing HSPA8 (Hsc70), DNAJB1 (class B J‐protein), and HSPH2 (Hsp110) (Gao *et al*., 2015). Here we show that reducing the Q35/44‐YFP aggregate load expressed in *C. elegans* muscle or intestinal cells requires a fully functional class A and class B J‐protein network. Distortion of this J‐protein collaborative function *in vivo* disrupts essential disaggregation activities and gives rise to the *in vivo* aggregate accumulations typical of protein misfolding pathologies observed in metazoan systems.

Unexpectedly, it is the constitutive housekeeping Hsc70 (HSP‐1) that is co‐opted *in vivo* with mixed‐class J‐protein complexes to form a highly regulated disaggregase, which is also supported by our *in vitro* findings. This indicates that Hsc70 is the relevant member of the Hsp70 family for recovering polypeptides from aggregates; no recovery when *hsp‐1* is knocked down. Lack of lifespan reduction in *hsp‐70*,* hsp‐70’,* and *hsp‐70’’* knockdowns relative to *hsp‐1* depletion further reflects this. It is also important to note that upon *hsp‐1* knockdown, expression of *hsp‐70* (C12C8.1) mRNA is more than sixfold upregulated (Guisbert *et al*., 2013), suggesting that a higher level of an inducible member of the Hsp70 family not efficiently compensate for loss of HSP‐1 activity in aggregate clearance. Taken together, these findings agree with the direct role reported for constitutive HSP‐1, but not induced HSP‐70 forms, in aging and age‐dependent pathophysiological conditions associated with protein aggregation in nematodes (Nollen *et al*., 2004; Brehme *et al*., 2014). A similar finding was recently reported for human HSC70 (HSPA8), which shows higher amyloid fibril disintegration activity compared to a stress‐induced HSP70 (HSPA1A) *in vitro* (Gao *et al*., 2015). The constitutively expressed HSPA8 constitutes by far the bulk fraction of Hsp70s in both unstressed and stressed human cells (Finka *et al*., 2015). Therefore, from an evolutionary standpoint, it is not surprising that the constitutively expressed isoform has selectively evolved as the primary metazoan Hsp70 for a general protein quality control function such as protein disaggregation. Identification of the divergent functions for different Hsp70 variants under stress, together with the threshold effects defined for Hsp70 and J‐protein efficacy during massive protein aggregation, suggests specific molecular ratios of protein quality control elements inside a cell (Manzerra *et al*., 1997; Chen & Brown, 2007) may be central to degenerative disease development. This opens new perspectives for consideration in the development of therapeutic strategies for protein conformational diseases.

In summary, this work defines a key regulatory J‐protein network, which under stress co‐opts the constitutive Hsc70 chaperone, rather than a stress‐induced variant, to rapidly generate nuanced and efficient *in vivo* disaggregation essential to whole animal survival. Insufficiencies in J‐protein class interactions and intracellular equilibria lead to a breakdown of Hsc70‐based disaggregation, aggregate accumulation, and drastically reduced viability in *C. elegans*. Our work establishes that cooperation among J‐proteins has an impact on organismal stress resistance, protein quality control systems, and the development of degenerative diseases, with potential broader relevance for the development of therapeutic approaches for other metazoans.

## Methods

### Plasmids and protein purification

*Caenorhabditis elegans dnj‐12*,* dnj‐13*,* dnj‐19*,* hsp‐110*,* hsp‐1*,* F11F1.1*,* C12C8.1,* and *F44E5.4* genes were amplified from *C. elegans* cDNA and cloned into pSumo vectors, and proteins were subsequently purified as described earlier (Nillegoda *et al*., 2015). Polyclonal antibodies against DNJ‐12, DNJ‐13, and DNJ‐19 were generated by immunization of rabbits with purified proteins (Charles River Laboratories, St. Germain‐Nuelles, France). The antisera were tested for specificity for their respective J‐protein by Western blot.

### Luciferase refolding and disaggregation/refolding assays

Luciferase disaggregation was performed as described previously (Nillegoda *et al*., 2015). In brief, thermal aggregation of luciferase was performed by incubating 0.025 μm of the native protein at 45 °C for 15 min in refolding buffer without the ATP regenerating system (40 mm HEPES‐KOH pH 7.5, 50 mm KCl, 5 mm MgCl_2_, 2 mm DTT, 2 mm ATP) in a water bath. The disaggregation reaction was started by adding 3 mm phosphoenolpyruvate, 20 ng μL^−1^ pyruvate kinase (ATP regenerating system) with the following concentrations of chaperones: HSP‐1, 8 μm; DNJ‐12, 4 μm; DNJ‐13 4 μm; DNJ‐19 4 μm; HSP‐110 4 μm, and by shifting the reaction to 20 °C. Each of the J‐proteins was set at 2 μm in the mixed‐class reactions. Final concentration of aggregated luciferase was set at 0.02 μm (monomer concentration). Luciferase reactivation was monitored at the indicated time points by transferring 1 μL of sample to 100 μL of assay buffer (25 mm glycylglycine, pH 7.4, 5 mm ATP, pH 7, 100 mm KCl, and 15 mm MgCl_2_) mixed with 100 μL of 0.25 mm luciferin.

### Sedimentation assay of luciferase

Nematodes expressing luci^R188Q,R216Q^‐YFP (luciferase designated as ‘luci’) were synchronized and subjected to heat shock for 1 h at 35 °C as L1s (1‐day‐old animals, 12 h posthatching) and subsequently shifted to 20 °C for recovery. Samples were collected before heat shock as well as 4, 12 h, and 4 days post‐heat shock. The nematodes were sedimented by spinning at 200 *g* for 2 min at 4 °C and washed twice with 0.1 m NaCl and the pellet was then flash‐frozen in liquid nitrogen. The nematode pellets were homogenized using a bead‐beating homogenizer (Precellys^®^24, Peqlab, Erlangen, Germany). Worm extracts were generated using ceramic beads at full speed (4100 g) for three times for 45 seconds. The cell debris was pelleted at 200 *g* for 5 min at 4 °C. The resulting supernatants were adjusted to identical protein concentration and subsequently centrifuged at 30 000 *g* at 4 °C for 2 h. The supernatant represents the soluble and the resulting pellet fraction the insoluble protein. The pellet was extracted in 50 mm Tris pH 8, 150 mm NaCl, 5 mm EDTA, 0.5% SDS, 1% NP‐40, 1 mm PMSF, Roche Complete Inhibitors and subjected to sonication (twice at level 2 duty cycle 65%). The protein samples were then subjected to SDS‐PAGE and Western blot analysis using monoclonal GFP antibodies (B34; Enzo Life Sciences, Farmingdale, NY, USA) to detect the luci^R188Q, R216Q^‐YFP protein.

### *Caenorhabditis elegans* strains and maintenance

Strains in this study were as follows: Bristol strain N2 (wild‐type), AM140 [*rmIs132 [*P*unc‐54::q35::yfp]*], AM141 [*rmIs297 [*P*vha‐6::q44::yfp+rol‐6(su10006)]*], and P*unc54::luci* ^*R188Q,R216Q*^ *::yfp*. Extrachromosomal P*dnj‐13*::*dnj‐13‐yfp* and P*dnj‐19*::*dnj‐19‐yfp* strains were obtained from Dr. K. Richter (TU Munich) and were integrated using gamma irradiation. Nematodes were grown on NGM plates seeded with *Escherichia coli* OP50 strain at 20 °C. Synchronization and RNAi experiments were performed as described earlier (Rampelt *et al*., 2012).

### Lifespan assay

Animals were plated as L1 onto fresh RNAi plates, subjected to a heat shock at 35 °C for 1 h as L1 or L4 larval stages (as indicated in figure legends), and immediately transferred back to 20 °C. In parallel, animals were maintained on the respective RNAi plates without heart shock. Animals were passaged every day onto fresh plates and monitored for survival from day 1 (L1 stage). Hundred animals were tested for each condition. Animals were considered dead if no movement was observed and if they did not respond to gentle prodding on the head.

### Fecundity assay

Animals were heat‐shocked as L4 at 35 °C for 1 h and immediately transferred back to 20 °C onto RNAi plates. The adult animals were transferred onto fresh plates every day until day 7 of life, and the number of eggs was counted for each animal over this period of time using a Leica MZ6 microscope. Ten animals were analyzed in parallel with one nematode per plate.

### Thermoresistance assay

Animals were maintained on RNAi plates at 20 °C until day 4. On day 4, animals were given 6 h of heat shock at 35 °C, then transferred immediately to 20 °C for 24‐h recovery and scored for survival using a Leica MZ6 microscope. Animals were considered dead if immobile and unresponsive to gentle prodding on the head. We analyzed 10 plates with 30 animals per plate.

Detailed methods for immunostaining, fluorescence microscopy, RNA isolation, cDNA synthesis, and qRT analysis are given in the Supplemental section.

### Immunostaining of nematodes

The integrated P*dnj‐13*::*dnj‐13‐yfp* and P*dnj‐19*::*dnj‐19‐yfp* strains were heat‐shocked for 1 h at 35 °C and subsequently shifted to 20 °C for 4 h before fixation with 4% paraformaldehyde for 30 min at room temperature. Fixed nematodes were then washed three times with PBS and then treated with a β‐mercaptoethanol solution (1 mL H_2_O, 400 μL 0.5 m Tris pH 6.8, 15 μL Triton X‐100, and 76 μL ß‐mercaptoethanol) overnight. The nematodes were then washed again with PBS three times and then incubated in 200 μL of collagenase solution (160 μL H_2_O, 40 μL 1 m Tris pH 7.4, 0.2 μL CaCl_2_, 4 μL collagenase stock solution (100 mg collagenase IV/mL 0.1 m Tris pH 7.4) at 37 °C under vigorous agitation for 30 min. The samples were placed on ice, followed by three washing steps using PBS and an additional washing step using Aba buffer (400 μL PBS, 5 μL Triton X‐100, and 10 mg BSA). The nematodes were then incubated with the primary antibodies against DNJ‐12, DNJ‐13, and DNJ‐19 overnight in a dilution of 1:200 each. The nematodes were washed three times with Aba buffer before the secondary antibody (Alexa Fluor 594‐conjugated goat anti‐rabbit IgG) was added in a dilution of 1:200 and incubated overnight. The nematodes were washed with Aba buffer and then mounted on glass slides with 1% agarose pads for subsequent imaging.

### Fluorescence microscopy of nematodes

For the analysis of the aggregation propensity of luci^R188Q, R216Q^‐YFP upon single and double knockdown of various *dnj* genes, 1‐day‐old P*unc54*::*luci* ^*R188Q, R216Q*^::*yfp* nematodes were heat‐shocked as L1 (1‐day‐old animals; 12 h posthatching) for 1 h at 35 °C and immediately returned to 20 °C and transferred onto RNAi plates. The aggregation propensity of luci^R188Q, R216Q^‐YFP was monitored prior to heat shock, 4 h post‐heat shock, and 4 days post‐heat shock. The aggregation propensities of intestinal Q44‐YFP and muscle Q35‐YFP were analyzed on days 6 and 5, respectively, with no prior heat shock. Animals were subjected to RNAi treatment from the first larval stage on and maintained on RNAi plates throughout the experiment. For imaging, nematodes were mounted onto 2% agarose (Sigma Aldrich, St. Louis, MO, USA) pads on glass slides and immobilized with 2 mm Levamisole (Sigma). Images were taken on a Leica SP5 or LSM780 confocal microscope at 20× and 63× magnification. The luci^R188Q, R216Q^‐YFP images were taken of muscle cells in the mid‐body region of the nematode. The Q35‐YFP‐expressing nematodes were analyzed as whole nematode for quantification of the aggregates, and an image was taken of the head region of the animal. For the intestinal polyQ model (Q44), images were taken of the complete intestinal tract of the animals. Twenty animals were analyzed for each condition. Aggregates were counted manually using a Leica M165FC stereofluorescence microscope and are defined as discrete, bright foci that can be distinguished from their surrounding fluorescence by increased brightness intensity as described previously (Silva *et al*., 2011). We employed ANOVA (Prism software), which is based on a pairwise comparison of the obtained data sets that tests the difference between two or more means, as the statistical method to analyze the aggregate quantification data.

### Fluorescence recovery after photobleaching (FRAP)

FRAP of Q35‐YFP was carried out as previously described (Garcia *et al*., 2007).

### RNA isolation and cDNA synthesis

Picked animals were snap‐frozen in liquid nitrogen. Total RNA was extracted with TRIzol reagent (Invitrogen/Thermo Fisher Scientific, Waltham, MA, USA), using 250 μL TRIzol and 50 μL chloroform. RNA from the aqueous phase was purified using NucleoSpin^®^RNA kit (Macherey‐Nagel, Düren, Germany) according to modified manufacturer's instructions. The quality of the isolated RNA was evaluated by agarose gel electrophoresis and measurements of the 260/280 as well as 260/230 nm absorption; 500 ng of RNA was reverse‐transcribed using Maxima First Strand cDNA Synthesis Kit for RT–qPCR (Thermo Fisher Scientific, Waltham, MA, USA).

### qRT–PCR

Quantitative RT–PCR was performed using Luminaris Color HiGreen High ROX qPCR Master Mix (Thermo Fisher Scientific, Waltham, MA, USA) in 20 μL reactions; 2 μL of a 1:10 diluted cDNA solution was analyzed in a two‐step cycling protocol according to the manufacturer's instruction of the Master Mix with a StepOne™ Real‐Time PCR System and StepOne™ Software version 2.0 (Applied Biosystem). Primers were designed by Primer3 software as previously described (Thornton & Basu, 2011) and tested for specificity, amplicon secondary structures, and primer structures using NCBI primer blast, the DINAMelt/Mfold Web Server, and Beacon Designer™ (Premier Biosoft, Palo Alto, CA, USA), respectively; 0.3 μm of the primers was used for the PCRs. Relative amounts of J‐protein mRNA expression were calculated with the 2^−ΔCt^ method (Livak & Schmittgen, 2001). Ct‐value duplicates were measured in three batches and averages of the duplicates were referred to the internal control *cdc‐42* to obtain ΔCt. Standard deviations were calculated from the ΔCt values of the three different batches. Sequences of the primers used in quantitative RT–PCR were as follows: *dnj‐12,* 5′‐GGTTCAACAAATGCAATCTCAC T‐3′ (forward), 5′‐CGTCTTCTTTCACCTGCTTCT‐3′ (reverse); *dnj‐19,* 5′‐GTGAG CCAGGAGATGTTGTC‐3′ (forward), 5′‐CAGTGATAGTTTCTTGGTCATGTG‐3′ (reverse); *dnj‐13,* 5′‐GAACTACAAGACGGCTGACT‐3′ (forward), 5′‐TGGATTGA GTTGTGATGGGA‐3′ (reverse); and *cdc‐42,* 5′‐AAACTTGTCTCCTGATCAGCT‐3′ (forward), 5′‐TACTGTG ACGGCGTAATTGT ‐3′ (reverse).

### RNAi and RNAi efficiency determination

RNAi knockdown was performed by placing about 100 L1 animals on NGM plates supplemented with 1 mm IPTG and seeded with *E. coli* expressing either the empty RNAi vector, L4440, or the genes encoding the respective *dnj* genes (Ahringer library, Source BioScience). Four‐day‐old animals were harvested for analysis of the efficiency of the knockdown. The RNAi efficiency was calculated by qRT analysis and indicated as percentage of the control (empty RNAi vector control). For double knockdown, the *E. coli* cultures expressing the RNAi clones were mixed with the same OD prior to seeding the RNAi plates.

### Co‐immunoprecipitation and Western blotting

A nonsynchronized culture of nematodes expressing luci^R188Q,R216Q^‐YFP was harvested on ice and flash‐frozen in liquid nitrogen. The pellet was then pulverized to powder in mortar on dry ice. The powder was then dissolved in lysis buffer (40 mm HEPES pH 7.4; 100 mm KAc, 5 mm MgCl_2_, 1 mm β‐mercaptoethanol, 2× Tm complete protease inhibitor (Roche), 0.1% Tween‐20) in low binding reaction tubes and then centrifuged at 200 rpm for 5 min at 4 °C. The supernatant was transferred to a new reaction tube, and the protein concentration was adjusted to 10 mg mL^−1^; 10 μL of each serum against DNJ‐12, DNJ‐13, or DNJ‐19 was diluted into 500 μL lysis buffer and was then incubated with 30 μL Protein A/G magnetic beads (Pierce) for 1 h at room temperature and then washed 3× with lysis buffer. The serum‐coupled beads were then incubated with 500 μL of protein lysate for 2 h at room temperature. Afterward, the beads were washed 4× with 500 μL washing buffer (40 mm HEPES pH 7.4; 150 mm KAc, 5 mm MgCl_2_, 1 mm β‐mercaptoethanol, 2× Tm complete protease inhibitor (Roche, Basel, Switzerland), 0.1% Tween‐20) and bound proteins were eluted with 1× SDS sample buffer. The samples were then subjected to SDS‐PAGE and subsequent Western blotting with primary antibodies against worm J‐proteins (1:5000 dilution) and secondary antibody anti‐rabbit HRP (1:10 000 dilution, Thermo scientific). The detection was carried out with the ECL Western kit (Pierce/ Thermo Fisher Scientific, Waltham, MA, USA).

## Funding

This work was funded by the Deutsche Forschungsgemeinschaft (SFB1036 and BB617/19‐3 to BB; SFB740 and NeuroCure Excellence Cluster EXC257 to JK), a postdoctoral fellowship of the Alexander von Humboldt Foundation (to NBN), the National Institutes of Health (NIGMS, NIA, NIMS), the Ellison Medical Foundation, HFSP, and the Daniel F. and Ada L. Rice Foundation (to RIM).

## Author contributions

NBN, BB, and JK conceived the study. NBN, JK, DLG, and BB designed the experiments. JK, NBN, KA, and ASc performed the experiments. NBN, JK, KA, ASc, ASz, DLG, RIM, and BB analyzed the data. NBN, JK, DLG, RIM, and BB wrote the manuscript.

## Conflict of interest

The authors declare there are no conflict of interest.

## Supporting information


**Fig. S1** Cooperative interclass J‐protein function for efficient disaggregation is precluded by triple knockdown of class A J‐proteins or mixed‐class J‐protein knockdown in heat stressed *C. elegans*.

**Fig. S2** Analysis of polyQ aggregation in *C. elegans* muscle and intestinal tissues in the presence of different J‐protein knockdowns.

**Fig. S3** Influence of RNAi knockdowns of Hsp70 disaggregase components on *C. elegans* lifespan.
