# Supplementary figures and images for: In vivo properties of the disaggregase function of J‐proteins and Hsc70 in Caenorhabditis elegans stress and aging
Source: Aging Cell. 2017 Oct 10;16(6):1414–24. doi: 10.1111/acel.12686 (PMC5676055; doi:10.1111/acel.12686)

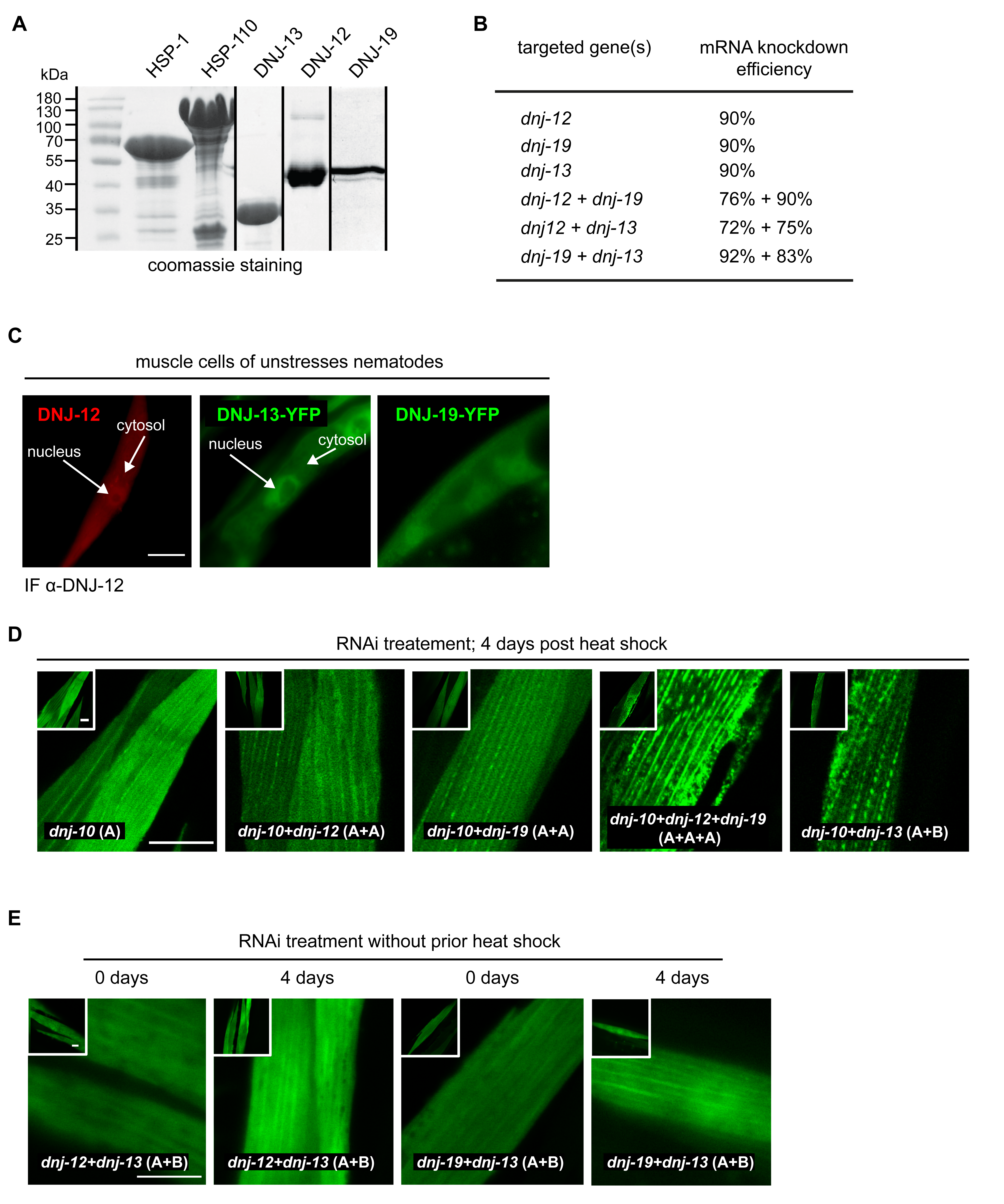

Supplement: Supplementary file 1 — Fig. S1 Cooperative interclass J‐protein function for efficient disaggregation is precluded by triple knockdown of class A J‐proteins or mixed‐class J‐protein knockdown in heat stressed C. elegans. [file ACEL-16-1414-s001.tif]

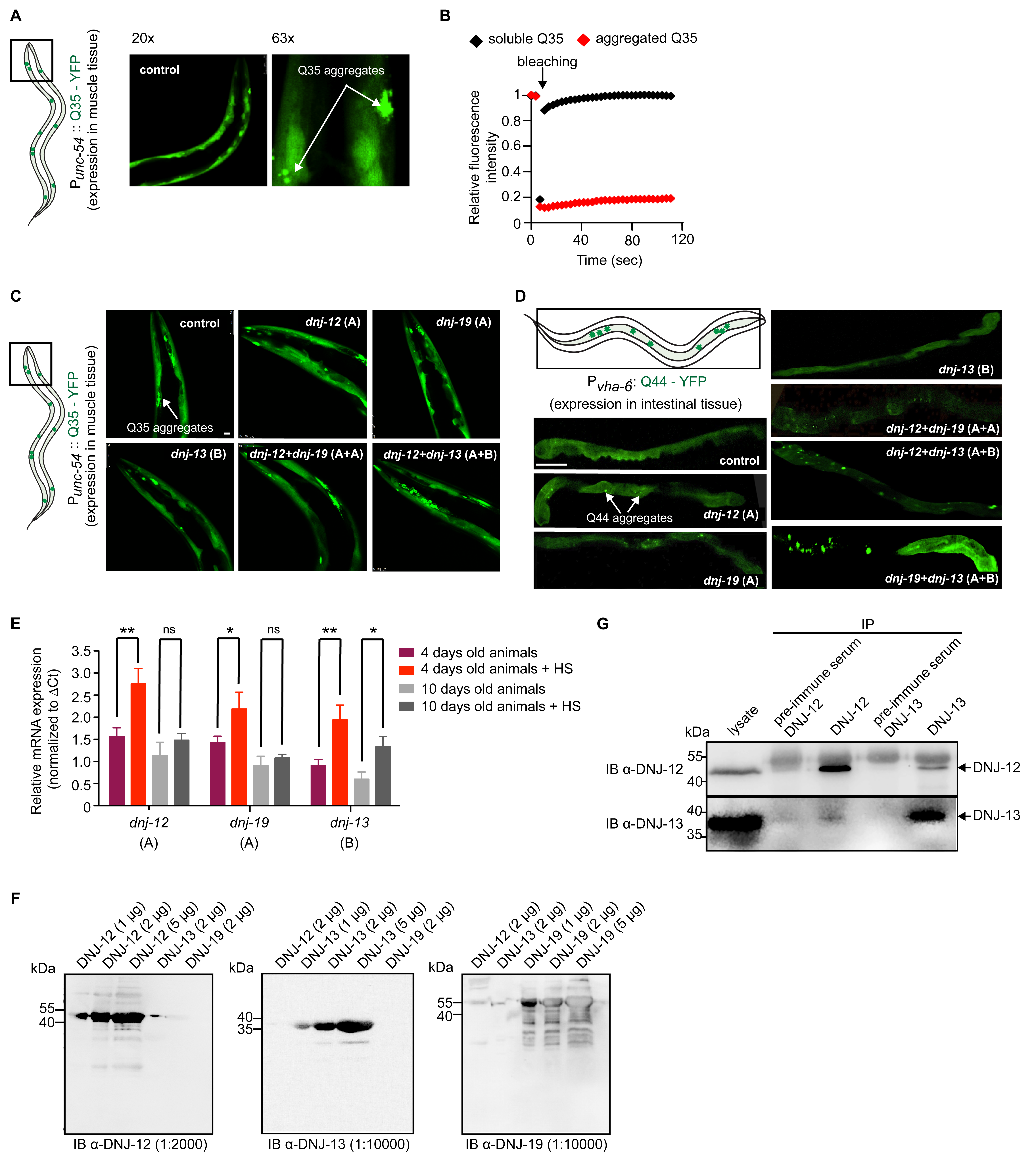

Supplement: Supplementary file 2 — Fig. S2 Analysis of polyQ aggregation in C. elegans muscle and intestinal tissues in the presence of different J‐protein knockdowns. [file ACEL-16-1414-s002.tif]

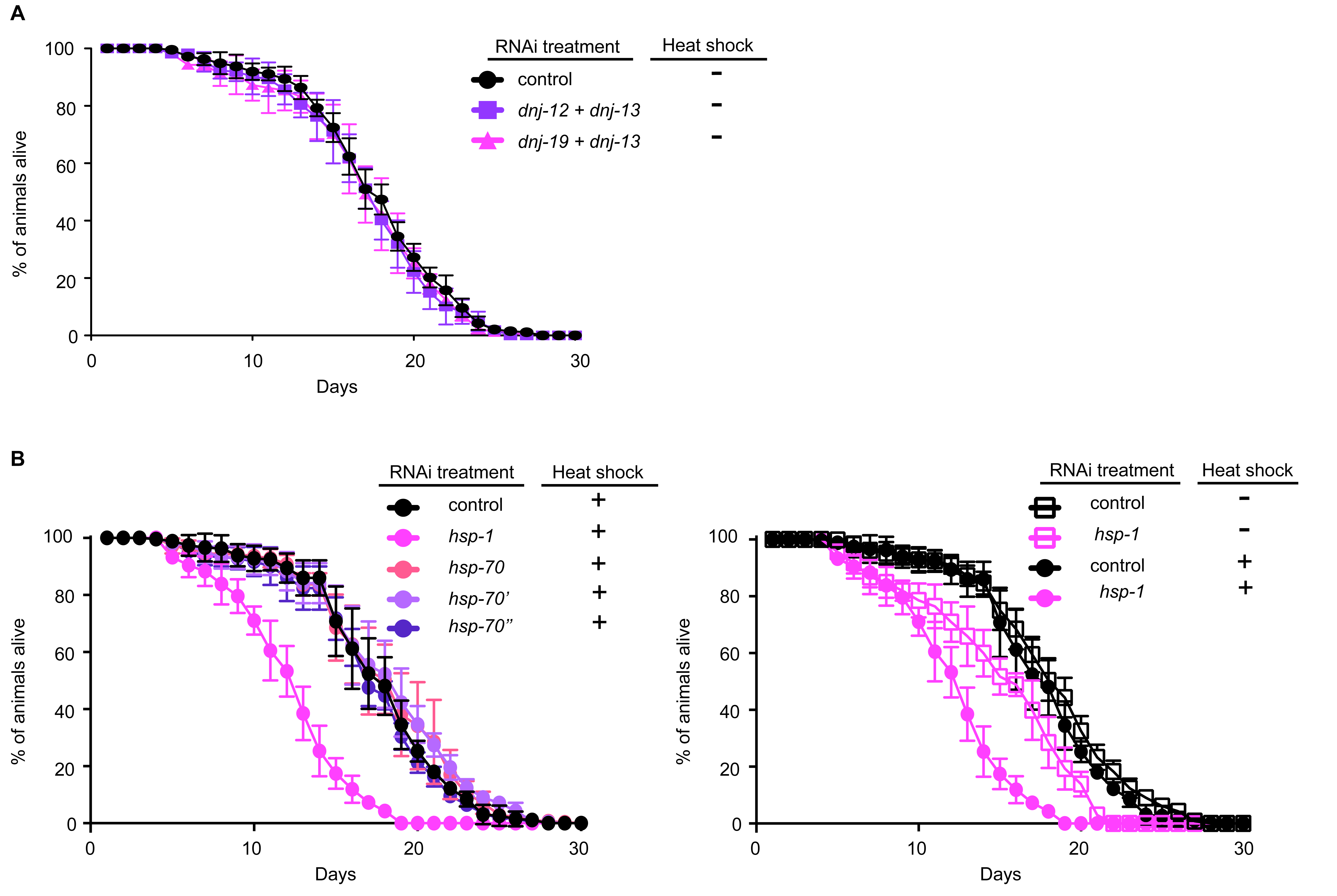

Supplement: Supplementary file 3 — Fig. S3 Influence of RNAi knockdowns of Hsp70 disaggregase components on C. elegans lifespan. [file ACEL-16-1414-s003.tif]
